# Exploring existing and deliberated community perspectives of newborn screening: informing the development of state and national policy standards in newborn screening and the use of dried blood spots

**DOI:** 10.1186/1743-8462-3-14

**Published:** 2006-12-13

**Authors:** Ian Muchamore, Luke Morphett, Kristine Barlow-Stewart

**Affiliations:** 1 IM Thinking Consulting, South Yarra, Victoria, Australia; 2 The Centre for Genetics Education, NSW Health, Royal North Shore Hospital, Sydney, NSW, Australia

## Abstract

**Objective:**

Since the 1960s newborn screening (NBS) for several rare and serious disorders has been in place across Australia. Testing of a simple blood spot now enables the early detection of over 30 conditions. Policies across Australian states have diverged in some aspects of NBS, especially in the retention and further use of dried blood spots collected as part of the screening and attempts are underway to bring some further national consistency. Whilst this has initiated debate amongst health professionals and policy makers there is limited empirical evidence of wider community attitudes to such issues.

**Methods:**

This research has explored the range and depth of views held by the wider community in New South Wales through moderated small group discussions. It has also assessed the range and depth of responses where the groups are reconvened after being given further information.

**Results:**

The findings suggest that there is limited community awareness of the public health importance of NBS and especially that resulting biological samples are stored. Members of the wider community presented with opportunities to consider current procedures and policies appear reassured and to have high levels of trust. However there are clearly some groups who have concerns with the storage of dried blood spot specimens and perceive that these may be abused.

**Policy implications and conclusion:**

The findings will inform health professionals and policy makers as to the perceived benefits and future challenges NBS raises for the wider community. The findings have implications for improving current communications about NBS, maintaining public confidence and the development of state and national initiatives in genetic health.

## Background

Newborn Screening (NBS) programs based upon blood samples from newborn babies have been operating in Australia since the late 1960s. The programs were initiated by the development of a biochemical assay by Guthrie to detect the chemical imbalances found in the blood of babies affected by phenylketonuria (PKU) [1]. Screening was expanded to include testing for congenital hypothyroidism, galactosaemia and cystic fibrosis [2,3]. The introduction in recent years of testing using Tandem Mass Spectrometry (TMS) methods [4] has enabled the inclusion of a much larger panel of conditions.

In the Australian state of New South Wales (NSW) the collected newborn blood spots are currently screened for about 30 conditions where early detection and treatment can positively impact on the health of an affected child and family. Annually about 85,000 babies born in NSW [5] are screened and about 90 babies are diagnosed with one of the screened conditions following a positive screening test. The blood sample, taken by heel-prick 48–72 hours after birth, is dried on cards originally known as Guthrie cards but which are now more appropriately referred to as dried blood spot specimens [6].

NSW Health policy [7] directs that informed verbal parental consent must be obtained before the procedure is carried out. The policy also requires that information is provided prior to testing in the form of a pamphlet [8] and that discussion should take place between health professionals and parents. It requires that consent is noted in the medical record of the mother and or child. Most parents do consent to screening and in cases where parents refuse NBS, policy requires that refusal is recorded in the mother's or baby's file. The parents are only informed of the test results from NBS if the result indicates a problem or further testing is required.

Screening programs in other Australian states have many similarities to NSW although there are some differences [2]. Whilst there has been some variation in the past as to precisely what conditions are tested for these are now essentially aligned apart from some technical differences in biochemical testing methods and their implementation. Protocols for collecting verbal consent vary slightly and there is also some variation in the content of educational information offered in advance to parents.

The clearest difference in approach has been in the retention policies for dried blood spot specimens which differ significantly across Australian states. Some states retain dried blood spot specimens indefinitely and others hold them for just two years before they are destroyed [9]. In NSW the dried blood spot specimens are stored for 18 years in a secure facility at the NBS Laboratory after which time they are destroyed [7]. The development of a uniform approach to newborn screening has been recommended by the Australian Law Reform Commission [9] and has also received support from the Australian Government [10].

The stored specimens may be used for quality assurance and audit practices by the laboratory and for the development of test protocols to further expand newborn screening. They may also be used, with the consent of parents, for the medical benefit of the family such as enabling prenatal testing in future pregnancies where information from an affected child is otherwise unavailable.

In addition, dried blood spot specimens may also be used for Human Research Ethics Committee (HREC) approved medical research [11]. Other uses include forensic testing. A formal agreement between NSW Health and NSW Police provides a protocol for police access to the specimens as part of their investigations [12].

Approximately 400 dried blood spot specimens from NSW have been used in the last 3 years for HREC approved research and about ten dried blood spot specimens since 2002 have been requested by the Police for the identification of remains of a person (personal communication Bridget Wilcken, Director NBS laboratory, NSW).

While NBS programs have been operating in Australia for over 40 years, the experiences, awareness, perceptions and attitudes towards NBS amongst the wider community have not been formally explored. In 2005 the Centre for Genetics Education undertook such a study exploring views about the program in NSW including the retention and further use of the dried blood spot specimens.

This paper focuses on consumer awareness and understanding of NBS and in particular views towards the retention and further use of the specimens. Responses to issues of consent, information provision and the potential to further expand NBS are being reported separately.

## Methods

A reconvened group discussion methodology, based upon previous studies exploring public perceptions of medical research [13], was used which allowed exploration of both existing and more deliberated viewpoints. The project and stimulus materials were developed with guidance from an Expert Advisory Group (EAG) with experience of NBS, policy, health ethics and communications. The research protocol was approved by the institution's Human Research Ethics Committee.

### Sample and recruitment

Discussions were undertaken with nine groups: 40 participants in total, comprising 24 women and 16 men (Table 1). Groups were structured such that participants shared similar socioeconomic backgrounds and were single sex to promote the development of group dynamics. It was intended to engage a cross section of distinct groups with potentially different stakeholder perspectives of newborn screening and the use of the stored dried blood spot specimens. The groups included young adults whose dried blood spot specimens had recently been destroyed, recruited through youth agencies in regional NSW (Groups 1–2); parents who had likely recently experienced NBS, recruited through several Child Health Centres in suburban Northern and Western Sydney (Groups 3–5) and parents with older children where the child's NBS died blood spots were being stored, recruited through a professional market research field recruitment agency in suburban Northern and Western Sydney (Groups 6–9).

**Table 1:** Sample Characteristics for Group Discussions

Group	Gender	No. Participants	Age Youngest Child	Area
1	F	8	-	Regional NSW
2	M	3	-	Northern Sydney
3	F	4	< 6 months	Inner West Sydney
4	M	3	< 2 years	Northern Sydney
5	F	3	< 6 months	North West Sydney
6	F	4	5 –18 years	Northern Sydney
7	M	5	5 –18 years	North West Sydney
8	F	5	5 –18 years	Inner West Sydney
9	M	5	5 –18 years	Inner West Sydney

Participants were provided with refreshments, childcare support where required and a $50 shopping voucher for attending both sessions.

### Discussion format

Each group met initially for about 90 minutes. Interviews were conducted using an agreed topic guide and by presenting stimulus materials intended to support further in-depth discussion. The discussions about NBS were introduced from a base of general health perceptions and information sources which then moved to understanding of the concepts of screening and testing in general. Whilst the research approach and use of the stimulus materials required some structure and order to the conversation, it was also possible to consider issues as they were naturally raised by participants.

Each group was reconvened between one and three weeks later. All but two of the younger women in Group 1 attended the reconvened sessions. These sessions were moderated in a less structured manner and focused upon the issues that participants themselves raised after having considered the issues and sought out the views of others in the community. The intention here was to examine how participants had considered the issues over time and to what extent their views had developed. Emphasis was placed upon discussions of the storage and further usage of the dried blood spot specimens.

Simple stimulus materials were developed to provide some focus for further discussion and deliberation around newborn screening and storage of the blood spot specimens. The materials covered current and potential future NBS testing; consent procedures and policies; the storage and further use of the dried blood spot specimens; and the rules governing access to them. The information was presented as several show-cards which covered the information contained in the NSW NBS pamphlet [8] in simple bullet-point format. Additionally four short scenarios were developed which were selected as examples covering the range of issues under deliberation:

1. A mother whose child is diagnosed with PKU

2. A couple overseas whose child is diagnosed with Duchenne muscular dystrophy

3. A couple who have been asked to consent to the further use of a stored dried blood spot specimen

4. A woman who wishes to access a dried blood spot specimen in a paternity legal action.

### Analysis

Group discussions were audio recorded and transcribed. Where possible the individual speaking within a group was identified in the transcript such that comments could be followed through each session and used in the analysis.

Transcripts were analysed using a Framework methodology [14] to identify and code common themes, using Microsoft Excel spreadsheets which allowed cross tabulation of individual responses against these themes. Where necessary, further development and editing of themes were undertaken during the coding process. Consistency in coding was validated by the authors independently coding several transcripts with differences discussed and resolved.

## Results

### Exploring Newborn Screening

For most participants NBS was not a familiar term by itself but they associated it with a number of newborn interventions of which they had some experience. The concept was also associated with other procedures such as vitamin K injections and Hepatitis B vaccination. There was little specific knowledge of protocols, procedures and rules related to NBS.

The key trigger for most participants who recalled the test was mention of the heel prick blood test rather than the term 'Newborn Screening'. The heel prick was recalled by most women with young babies and with older children and was also reported by all the younger fathers; a few of the fathers with older children also had quite distinct recollections of the test being done. In particular parents recalled whether they had been present at the time of the test and whether the baby cried after the heel prick. Another common recollection was that this was the test where parents were only informed of the results if there was some problem.

Recollection of the heel prick was not generally linked to an understanding of what specifically was being tested for but this did not seem a cause of concern. One father who had videotaped his child having a heel prick stated: "*I don't know what they test for. I know it's something good. It's not bad*" ^Group4a^. Some participants suggested that the heel prick was part of testing for conditions such as autism, HIV, jaundice or blood clotting diseases. A few participants seemed more confident in their knowledge and stated that the heel prick test was for cystic fibrosis. Two individuals recalled that the test was in some way linked to the *"artificial sweetener condition" *[PKU]^Groups4a,5a^. In one case this information was gleaned from reading the label on the can of a diet soft drink. The other participant (a woman with several family members in the medical profession) specifically mentioned that one of the conditions included was called PKU and that individuals affected needed to follow a special diet. NBS was generally unfamiliar to the two groups of younger people (Groups 1–2) even when prompted by the term "heel prick".

### Support for Newborn Screening

All participants were highly supportive of the principles of current NBS as outlined in the show-card presented to them. Early detection of a condition was a key positive aspect for them. They knew little about the specific conditions detected and were surprised to hear that as many as 30 different conditions could be detected by NBS. A few participants were keen to have further information about these other unfamiliar "*alphabet diseases*" ^Group9a, Group9b ^currently included in NBS. Participants often did not initially realise that many of the conditions detected were inherited in families.

When presented with the first scenario which covered current PKU screening their responses were overwhelmingly positive. None of the participants raised personal or specific concerns with testing for PKU. Participants assumed that almost all parents would see the value in screening for such a treatable condition. Most also reacted positively to the further expansion of screening to include additional conditions, such as Duchenne muscular dystrophy, where there are more limited, if any, options available. At the same time, there was recognition of the impact of such an early diagnosis on parenting and the family. Further analysis of the discussion surrounding the conditions included in NBS and information provision is being separately reported.

### Storage of the dried blood spot specimens

Participants were prompted to discuss their wider experience, knowledge and views towards healthcare related to tissue and blood samples. Such understanding was limited, with almost all believing that such health samples would be destroyed after initial testing. Common reasons cited were that storage would serve no purpose; the sample is no longer needed; the patient can just give another sample if it is necessary and the results are probably kept but physical samples would be impractical to store. On probing and with further reflection a few suggested that there could be some value in storing certain samples, especially tissue for further testing and medical research.

All participants were initially unaware that the dried blood spot specimens, including those of their own children, were being stored. When presented with this information on a show-card, reactions included surprise, curiosity and even shock. It immediately raised general questions such as *"What's the point of storing these blood spots?"*, *"Why the 18 years?" *and *"Wouldn't such widespread storage be cumbersome*?" Considering this new knowledge that the dried blood spot specimens were stored led some to begin considering what had happened to other health samples that had been taken from them.

Participants also found that the friends and family with whom they spoke between the discussion sessions were equally unaware that the dried blood spot specimens were kept. Only one participant found someone who was already aware that they were stored: their partner had seen a national television show that included some discussion about the storage of the specimens. Almost all participants felt that it was vital for parents to know that the dried blood spot specimens are stored for 18 years and potentially used for various purposes.

### Using the dried blood spot specimens

When asked to further consider and speculate just why they thought the dried blood spot specimens were stored, unprompted responses included that they could be used for quality assurance or re-testing. Some participants felt they could be useful for the family to find a reason for an illness that had later affected their child. Some considered it would be possible to extract DNA from them. Others had not previously made this link but as they considered what they knew about DNA forensics from popular television dramas it was clear to them this was indeed a possibility.

#### (a) 'Family'

The possible use of the dried blood spot specimens by families was often interpreted as parents being able to access them at a later date for further health information about their children. This included exceptional circumstances such as using them to later investigate why a child had died.

More commonly there was the perception that the stored specimens might provide some direct benefits to the child or family. It was suggested that parents may desire further tests on the specimens as their child grew up and that taking another blood sample from the child could be avoided by accessing them. Concern was expressed among some of the younger women whose specimens had recently been destroyed that they would 'miss out' on these perceived personal and family benefits.

#### (b) Medical research

Use in medical research was generally viewed supportively. It was widely agreed that parents should be informed and consulted if identifying information would be provided to researchers requesting the dried blood spot specimens. The concept of research upon anonymised samples was clearly grasped and seeking parental consent in such cases only viewed as important by a few. A couple of individuals in different groups came up with the suggestion that the samples could be permanently anonymised at 18 years by simply cutting off the identifying information from the card and this could extend the life of this apparently valuable research resource.

Unacceptable uses of the dried blood spot specimens for research commonly included human cloning and any research that might lead to discrimination by insurers or employers. Some had strong views that access by pharmaceutical and biotechnology companies should be resisted, views that were underpinned by a belief that health research and developments should not be driven by a profit principle. It was also argued that commercial companies might bias or manipulate research findings. Such suggestions prompted others to counter that advances in health would not happen without such commercial motives. The idea of more commercially oriented research was often more palatable if there could be some clear return of benefits to the community or public health system.

Whilst participants generally agreed that there would be some research areas and situations they would find problematic, they were generally prepared to let these decisions lie with others representing the public good such as research ethics committees. The concept and role of such bodies was also easily grasped and even though little was known about their workings a high level of trust and support was expressed for their judgments.

#### (c) Access by police and courts

Access to the samples by the police for identifying missing persons and solving crimes was not controversial for many participants and several saw considerable potential value in the stored samples to identify missing children. They cited media stories published during the fieldwork period which documented attempts to identify a missing child using DNA testing [15]. Popular television shows focusing on forensics were often mentioned as a source of knowledge about the use of DNA from blood samples in identifying human remains, missing persons and in solving crimes.

Several participants also viewed positively the potential to use the dried blood spot specimens in some manner to identify a suspect in a crime and for many it was a case *of "if you have nothing to hide what would the problem be?" *^*Group 3b*^. However in each group of men some concern was expressed about the behaviour of the police and potential misuse of the dried blood spots. Those men who expressed such concerns demonstrated a clear general lack of trust in the police. While outlining the current agreement in NSW between police and health authorities appeared to be reassuring, there was still some continuing concern expressed among several of the male participants. Even if the blood spot sample was released initially for apparently legitimate reasons these men suggested it might later be used for other illicit purposes including such as by corrupt police officers to frame an individual.

Scenario four raised the potential use of samples for paternity testing and the involvement of the courts stimulated some further discussion. The justice system was seen as the appropriate mechanism through which requests for access to the specimens in cases such as this should proceed. A common response was that if the court demanded access to the dried blood spots for testing, little resistance could be made.

#### (d) Retention of dried blood spot specimens, ownership and records

A common initial response to learning about the storage of the dried blood spot specimens was to query how practical this was and the space requirements it raised. Questions were raised as to how securely access was controlled and it was suggested that no system could ever be impenetrable to abuse. A number of participants, mostly men, cited media coverage of the inappropriate use of personal information by police, other government agencies and private companies which illustrated how public privacy concerns had increased in recent years. Privacy concerns such as identify theft, credit card fraud and problems with internet security highlighted to them that storing the dried blood spot specimens raised privacy and security issues. Several male participants suggested unprompted that the samples could be used to create a DNA database.

The rationale for the 18 year storage period for the NSW dried blood spot specimens perplexed several participants. While several participants suggested that they be stored for longer, others suggested that the current cut off was likely explained in some way by the child reaching maturity and therefore fresh consent might need to be sought.

A common question posed was why the specimens were not stored for longer if they had such value for further medical research. Based on what they had heard and learnt in the group discussions most were prepared to accept the existing position of 18 years. No participant suggested that currently stored dried blood spot specimens should be destroyed prior to the current 18 year retention period.

Generally ownership of the specimens was not claimed by participants although they did view that they had some rights over them and should have some say over how they were used. A distinction was made by several participants between the storage of the specimen itself and the records containing the results derived from testing. They questioned whether the electronic records were also destroyed after 18 years.

In the reconvened groups moderators raised the fact that there were different storage retention policies in different Australian states. The variation was surprising and unsettling for many participants. They had expected that the NBS policies and issues they were discussing would be similar across Australia. The fact that the retention periods differed so significantly led them to question if there was further information available which might provide a rational explanation.

Concerns were also expressed if an individual relocated to another Australian state it may be difficult for them to access the stored dried blood spot if ever necessary. Such comments were in the context of the perceived direct personal benefit from the storage. They questioned in such cases if it was possible for the specimens to be transferred between states.

## Discussion

Participants in this study had some vague initial understanding of the procedures of NBS, yet they knew very little about its specific purpose and implications. There would also seem to be a very low level of community awareness that the dried blood spots are stored and indeed many people do not seem to have previously considered what happens to other biological samples they have given in health settings.

While the concept of accessing the dried blood spots for other uses was new to the groups, all quickly understood the issues and were able to participate in the discussions. Despite the surprising discovery that these dried blood spot specimens were kept, there was no evident rush to demand they should be destroyed because they were unaware of their existence. It appeared that this high level of community trust and support for storage was underpinned in part by a misconception that the specimens could *commonly *have direct future personal benefit to an individual. It was evident that when participants were discussing testing as part of NBS, they were also often considering that the dried blood spots might be used for additional tests at a later date in a child's development. In reality examples of benefits for the family, where they occur, are in enabling prenatal testing and for forensic identification.

The support for the use of the dried blood spot specimens in medical research may be reassuring. All participants grasped the distinctions between identified and anonymised research and seemed satisfied with the rules and regulations as they were presented to them. This study found very high levels of trust in the medical profession and government regulation. There was very little questioning of the mechanisms in place to oversee research upon samples and rather participants were accepting that if an ethics committee is in place it can be trusted to act in the public good. There was little evidence of the mistrust and cynicism with scientists and institutions that has been described in some European Countries [16].

Support amongst participants for use of dried blood spot specimens in identifying missing persons was particularly strong. Linked to this view were the concerns raised by young people whose specimens had recently been destroyed about the loss of utility for them in this context. There was some support in this study for the use of dried blood spot specimens by police to identify suspects in criminal cases. Findings from international public surveys [17] also suggest that many members of the public, at least in the UK, would express support for this use. While this general support for access to the cards by the police indicates that many may view this positively, it was also very clear that there were a number of participants strongly objected to access by such third parties. Trust, or lack thereof, seemed to underpin these views as well as the experiences of some of the participants, particularly men, in the use of electronic data. Concerns were also expressed about the privacy and security of the samples, the possibility of identity fraud as well as inappropriate use of data and forensic samples by the police.

Whilst many in the community may have high levels of trust this is likely to be impacted by critical media coverage of a NBS program. In this context it is noteworthy that, to date, there has been little media coverage, of any form, related to NBS in NSW. A search, using an online catalogue, for recent media articles about NBS revealed almost nothing in NSW yet several stories raising concerns about the dried blood spot specimens were found from other Australian states and also from New Zealand [18,19]. Some health professionals in other Australian states have suggested that the concerned media and "conspiracy theorists" should be reassured by the strict controls that already exist to protect information [20]. However it is also clear that it is better to consider and address at the outset issues which are likely to be sensitive or have the potential to raise controversy in the community. Once lost public trust can be very difficult to rebuild and in part it needs to be maintained by listening to public concerns seriously, even if they appear at first irrational, and by considering what underlies them [16].

The qualitative approach used here presents opportunities for extended discussion, consideration of information and further deliberation over a few days. It therefore supports a deeper exploration and understanding of community views than some other methodologies. It is evident from our findings that a quantitative approach, such as implementing a survey, would be of limited initial value in engaging these stakeholder groups in policy issues. Whilst many people would probably give a response to a survey question, if they do not understand the question and the background, some responses may simply be 'non-opinions'.

Public opinion polls and surveys do have a role in informing health policy development in this area. A recent mail survey of mothers from Western Australia generally confirms the picture we have found in that many parents do not feel well informed about NBS and storage of the dried blood spot specimens [21]. Nevertheless the survey findings also highlighted some of the methodological difficulties in engaging consumers and assessing their views. When asked in the survey, almost one in three mothers supported the current two year retention for dried blood spot specimens. However these mothers also noted that they did not possess adequate knowledge of the issues to consider any alternative options for retention policies and therefore supported the *status quo *as presented to them. It is in situations such as this that there is significant value in a more deliberative approach to exploring valid community views. In contrast to the Western Australian study, we did not discern broad concern for the extended storage of the dried blood spots. This significant difference may reflect the background and experience of the participants as well as the more deliberative consideration of the issues enabled by the methodologies used in our study. The findings from the study presented here provide a basis for further research exploring any differences in perspectives of the wider Australian community.

All methodologies which attempt to explore and consider public attitudes and opinion have their own limitations. Whilst the qualitative approach used here was designed to capture a range of different perspectives it does not claim to present a representative picture of the population's views. This study included few people from culturally diverse backgrounds or other groups whose views may be quite different from those reported. It is worth noting that several studies in other countries suggest that those from certain ethnic minority backgrounds are much more likely to have concerns with the storage of biological samples, access to genetic databases by third parties and lower level of trust in health providers [17,22]. We would expect there to be some further sensitivities towards NBS programs amongst these and other vulnerable groups.

Furthermore in this study discussions about NBS and associated issues of storage and further use were framed in the context of health, testing and screening generally. NBS was presented to participants in a medical context which initially highlighted its direct application in detecting rare and treatable conditions. It was on this canvas that further discussion was initiated. Many participants seemed to find it difficult to move beyond the immediate perceived personal benefits NBS testing and dried blood spot specimen storage could offer them.

Genuine community consultation is essential to support attempts to develop national policy standards especially in areas which are currently of greatest divergence, notably the retention and further use of dried blood spot specimens. It will require an understanding of community perspectives and their involvement in building a model which has widespread support. It will also likely require some further development and testing of appropriate public involvement methodologies.

## Policy implications and conclusion

This deliberative and consultative process has provided rich data to inform the development of state and national policies in NBS. It especially provides a deeper insight into community views of the retention and storage of dried blood spot cards. The findings suggest that, with the provision of information and opportunities for deliberation, many members of the wider community are likely to support the extended storage of dried blood spot specimens and their use in medical research.

The printed materials offered to parents in NSW [8] are notable in that they explicitly outline that the blood spot cards are stored and how they may be accessed and used in the future. However it would seem that many parents fail to absorb or are aware of this information. In line with the findings of this study NSW Health NBS policy has recently been updated and now includes further advice for health professionals to effectively communicate to parents that the blood spots cards are stored [7].

There is additional evidence that community awareness of NBS may also be limited in other Australian states [23]. Researchers conducting a community study commissioned by the Victorian Government [24] have suggested that parents are not given enough information about newborn screening and that there is a general lack of understanding about storage procedures [25]. This research has informed the development of a policy review which recommends to the Victorian Minister for Health an improved community education program and that extended storage of the blood spot cards should include explicit written consent from parents [26].

These policy issues potentially have national as well as state relevance. The consternation expressed amongst those we spoke to regarding differences in policies for NBS across Australia supports the current deliberations of the Australian Health Ministers' Advisory Committee where a subgroup has been asked by the Commonwealth Government to consider the development of national NBS standards [10]. In our study the different State NBS policies regarding storage of dried blood spot cards were identified as a matter of concern that required rationalization. One aspect participants highlighted was that family members, often dispersed geographically, should have equitable access to the perceived benefits in storing the dried blood spots.

This study demonstrates that members of the wider community are clearly able to engage and consider some of complex issues and grasp some of the key scientific, social and ethical issues raised by developments in genetic health. These participants were not specialists in health, policy or ethics yet they were able to contribute unique perspectives as consumers of genetics services. Those we spoke to embraced the sometimes challenging and complex issues, considered these carefully and were willing to share and listen to views of others. They were also willing to problem solve and make positive suggestions as to how policies and practices could be further developed. This bodes well for a more inclusive strategy in the development of public policies in the developing field of genetic heath.

## References

[B1] Guthrie R, Susi A (1963). A simple phenylalanine method for detecting phenylketonuria in large populations of newborn infants. Paediatrics.

[B2] Wilcken B (2003). Does every baby get a newborn screening test?. Medical Journal of Australia.

[B3] Crossley JR, Elliott RB, Smith PA (1979). Dried-blood spot screening for cystic fibrosis in the newborn. Lancet.

[B4] Wilcken B, Wiley V, Hammond J, Carpenter K Screening newborns for inborn errors of metabolism by tandem mass spectrometry. New England Journal of Medicine.

[B5] Centre for Epidemiology and Research (2005). NSW Department of Health. New South Wales. Mothers and Babies 2004. NSW Public HealthBull.

[B6] International Society for Neonatal Screening, Standing Committee on Quality Assurance (2005). Lexicon of terms to be used in newborn screening.

[B7] NSW Department of Health. New South Wales (2006). Newborn Bloodspot Screening Policy, Policy Directive PD2006_099.

[B8] NSW Department of Health. New South Wales (2004). Tests to Protect Your Baby.

[B9] Australian Law Reform Commission (2003). Essentially Yours: The Protection of Human Genetic Information in Australia, ALRC 96 Canberra.

[B10] Australian Government. Attorney General's Department (2005). Australian Law Reform Commission and Australian Health Ethics Committee Report Essentially Yours The Protection of Human Genetic Information in Australia Government Response to Recommendations Canberra.

[B11] National Health and Medical Research Council (1999). National Statement on Ethical Conduct in Research Involving Humans Canberra.

[B12] NSW Department of Health. New South Wales (2002). Memorandum of Understanding between The New South Wales Commissioner of Police and Director General of the New South Wales Department of Health.

[B13] The Wellcome Trust (1998). Public Perceptions of Human Cloning A Social Research Study London (UK); ISBN 186983500X.

[B14] Swallow V (2003). Research Brief. How to manage and display qualitative data using 'Framework' and Microsoft Excel. Journal of Clinical Nursing.

[B15] King D DNA Test For Schoolgirl Who Could Be Baby Tegan. The Australian.

[B16] House of Lords Select Committee on Science and Technology (2000). Third Report, Science and Society UK.

[B17] Human Genetics Commission (2001). Public Attitudes To Human Genetic Information People's Panel Quantitative Study UK.

[B18] Macleod S Third parties increasingly access material from blood bank DNA. New Zealand Herald Auckland (NZ).

[B19] Giles T, Kelly J Baby DNA confusion. Parents unaware of gene bank. Herald-Sun Melbourne.

[B20] Skene L, Bankier A (2004). Retention of Guthrie Cards: Reassuring Parents. Medicine Today.

[B21] Davey D, French D, Dawkins H, O'Leary D (2005). New mothers' awareness of newborn screening, and their attitudes to the retention and use of screening samples for research purposes. Genomics, Society and Policy.

[B22] The Wellcome Trust, Medical Research Council (2002). Public Perceptions of the Collection of Human Biological Samples Qualitative research to explore public perceptions of human biological samples UK.

[B23] Suriadi C, Jovanovska M, Quinlivan JA (2004). Factors affecting mothers' knowledge of genetic screening. Australian and New Zealand Journal of Obstetrics and Gynaecology.

[B24] State Government Victoria, DOHR (2005). Request for Quotation Report into Informed Parental Consent for Newborn Screening in Victoria.

[B25] Health Issues Centre Response to the Australian Health Ministers' Advisory Council (AHMAC) Advisory Group's Consultation Document (Principles and Guidelines for Newborn Screening – a uniform approach to newborn screening based on bloodspots for Australia).

[B26] Victorian Government Department of Human Services (2006). Victorian Newborn Screening Review Committee Final report for the Minister for Health.

